# An Ensemble Deep Learning Model with a Gene Attention Mechanism for Estimating the Prognosis of Low-Grade Glioma

**DOI:** 10.3390/biology11040586

**Published:** 2022-04-12

**Authors:** Minhyeok Lee

**Affiliations:** School of Electrical and Electronics Engineering, Chung-Ang University, Seoul 06974, Korea; mlee@cau.ac.kr

**Keywords:** survival estimation, prognosis estimation, deep learning, attention mechanism, gene expression, low-grade glioma, *HILS1*

## Abstract

**Simple Summary:**

This paper proposes a deep learning model for prognosis estimation and an attention mechanism for gene expression. In the prognosis estimation of low-grade glioma (LGG), the proposed model, Gene Attention Ensemble NETwork (GAENET), demonstrated superior performance compared to conventional models, where GAENET exhibited an improvement of 7.2% compared to the second-best model. By the proposed gene attention, *HILS1* was discovered as the most significant prognostic gene for LGG. While *HILS1* is classified as a pseudogene, it functions as a biomarker for predicting the prognosis of LGG and has been shown to have the ability to regulate the expression of other prognostic genes.

**Abstract:**

While estimating the prognosis of low-grade glioma (LGG) is a crucial problem, it has not been extensively studied to introduce recent improvements in deep learning to address the problem. The attention mechanism is one of the significant advances; however, it is still unclear how attention mechanisms are used in gene expression data to estimate prognosis because they were designed for convolutional layers and word embeddings. This paper proposes an attention mechanism called gene attention for gene expression data. Additionally, a deep learning model for prognosis estimation of LGG is proposed using gene attention. The proposed Gene Attention Ensemble NETwork (GAENET) outperformed other conventional methods, including survival support vector machine and random survival forest. When evaluated by C-Index, the GAENET exhibited an improvement of 7.2% compared to the second-best model. In addition, taking advantage of the gene attention mechanism, *HILS1* was discovered as the most significant prognostic gene in terms of deep learning training. While *HILS1* is known as a pseudogene, *HILS1* is a biomarker estimating the prognosis of LGG and has demonstrated a possibility of regulating the expression of other prognostic genes.

## 1. Introduction

Owing to the rapid development of deep learning algorithms, artificial intelligence models based on deep learning have become mainstream in various domains [1,2,3]. In biomedicine, such models have been extensively studied for medical imaging [4,5,6], diagnosis [7,8,9], and genome sequencing [10,11,12]. The prognosis of diseases also has been estimated with deep learning models in recent studies [13,14,15]. Generally, clinical data, such as age and sex, have been used in those models to estimate the prognosis [16,17].

Recently, gene expression data have been regarded as an alternative approach for estimating prognosis since gene expression is highly related to the prognosis of various diseases [18,19,20]. For these reasons, deep learning models handling gene expression data for estimating prognosis have been proposed in several studies [21,22,23]. For instance, Wong et al. [21] proposed a deep learning model for discovering prognostic genes related to glioblastoma. They introduced partial likelihood that signifies survival risk as a loss function to train a deep learning model called multilayer perceptron (MLP). Chaudhary et al. [22] employed multi-omics gene expression data with RNA sequencing, miRNA sequencing, and methylation data to train a deep learning model for the survival prediction of hepatocellular carcinoma. An auto-encoder (AE) deep learning model was introduced in this work.

However, these studies have commonly used basic deep learning architectures, such as MLP and AE [21,22]. In recent years, various superb deep learning architectures have been proposed for computer vision and natural language processing, resulting in the rapid advance in such domains [24,25,26]. While not all these architectures and methods can be applied to the prognosis estimation with gene expression data, it is true that it has not been extensively studied to address such advances thus far.

The attention mechanism is one of the recent advances in deep learning architecture, which has been widely used in computer vision and natural language processing [27,28,29]. An attention layer regularizes feature maps and word embeddings to make deep learning models more focused on specific regions related to the targets of the models, whereas the other regions are multiplied by approximately zero; thereby, the layer produces valid values only for the specific regions. The attention layer can be successfully adopted for computer vision and natural language processing because, in images and natural language, there are definite regions that determine the estimations. For example, in object detection in an image, an area where a particular object exists becomes the specific region on which the attention layer focuses [30,31].

Similarly, in gene expression data, it is considered that there are specific genes related to prognosis and survival, and the other genes are less associated with prognosis [32,33,34]. Therefore, it is expected that deep learning models with gene expression can enjoy the advantages of the attention mechanism. Since attention layers can learn which genes are related to the target (i.e., prognosis), it is also possible to determine prognostic genes with a nonlinear perspective of deep learning. However, the conventional attention mechanism cannot be directly applied to a deep learning model with gene expression because existing attention layers commonly use convolutional operations and multiplications between word embeddings [27,28,30], which cannot be done in models for gene expression.

This paper proposes a modified attention mechanism for gene expression, namely gene attention, to address this limitation. A gene attention layer using the attention mechanism is employed in the first layer of the model. In the gene attention layer, prognostic genes are determined by learning from data with a deep learning architecture. Then, high weights are multiplied for estimated prognostic genes, whereas the other genes are multiplied by low weights, approximately zero. In this process, it is possible to enhance the efficiency and accuracy of the proposed estimation model; additionally, the gene attention layer identifies which gene is associated with the prognosis and survival, which corresponds to one of the advantages of the attention mechanism.

Furthermore, recently developed deep learning architectures, which are now conventional in computer vision and natural language processing, are employed in the proposed model. The residual learning scheme [35] is adopted to enable the training of a deep architecture; layer normalization [36] is employed for smooth gradient flows in the backpropagation of the deep architecture; the ensemble learning method [37] is used to prevent overfitting and generalize the estimations of the model.

As the gene expression for the input variables of the proposed model, RNA sequencing (RNA-seq) data are used. Given that the transcriptome generates proteins, and proteins define the phenotypes of patients, it is essential to consider the transcriptome of patients in order to predict prognosis. RNA-seq is a technique for expressing the transcriptome in an organism that has been widely employed in recent years. While a few studies have introduced multi-omics, which utilizes additional types of transcriptome such as miRNA, or multi-modal schemes, which employ medical images [15,22], in practice, it is highly uncertain that such multiple data types can be simultaneously obtained for each patient. These models with multi-omics or multi-modal schemes cannot perform the estimation if any of the data types used in the models is not obtained.

In this paper, the prognosis of low-grade glioma (LGG) is estimated by the proposed model. Generally, without appropriate intervention treatments, LGG is eventually aggravated to high-grade glioma (HGG) and leads to death [38]. Therefore, it is of great importance to estimate the prognosis of LGG in order to determine if urgent treatment is required. For instance, it is possible to minimize side effects and treatment costs by prescribing moderate treatments to patients with a favorable estimated prognosis, while extensive treatments may improve survival rates for patients with a poor estimated prognosis. Additionally, it has been reported that the prognosis of LGG is highly correlated with gene expression compared to other cancers [39]; thus, the prognosis of LGG can be a suitable target to verify the performance of the model.

## 2. Materials and Methods

### 2.1. Deep Learning with Gene Attention

A conventional deep learning model consists of multiple layers, each of which has a weight matrix and a nonlinear function. In each layer, the input values are multiplied by a weight matrix, and then a nonlinear operation is performed [40]. The result values become the input values of the next layer. This process continues to the final layer. Appendix A describes the fundamentals of deep learning models in further detail. The conventional deep learning model can be summarized by the following equation:

(1) $$\hat{Y}\;≔\;\sigma_{N}(W_{N}\cdot \sigma_{N-1}(W_{N-1}\cdots \sigma_{2}(W_{2}\cdot \sigma_{1}(W_{1}\cdot X)))),$$

where $\hat{Y}$ is an estimation vector by the deep learning model; $\sigma_{k}$ and $W_{k}$ are the nonlinear function and the weight matrix for the kth layer, respectively; X is an input vector of the model; N is the number of layers in the model; all bold terms in the equations represent a matrix.

The attention mechanism corresponds to multiplying specific values to the layer outputs [27]. Generally, an attention value from zero to one is multiplied by each output feature. The attention value indicates how much the corresponding feature is delivered to the next layer. For instance, if an attention value of one is used, the corresponding feature is fully conveyed to the next layer, whereas information in a feature would not be transferred if the value is zero. This process in the kth layer can be represented by the following equation:

(2) $$X_{k+1}≔A^{T}⊙\sigma_{k}\left(W_{k}\cdot X_{k}\right),$$

where $X_{k}$ is the intermediate output from the k−1th layer; A is an attention vector containing the attention values for each feature; ⊙ denotes component-wise multiplication. The attention values are also determined by learning from data. The attention layer produces the attention vector A and has a weight vector to be trained; it is simultaneously trained with the other deep learning architecture by the backpropagation algorithm to estimate the target.

Various structures exist according to datatypes and deep learning architectures to implement the attention layer. However, most structures are developed and used for a convolutional layer for images as well as embeddings of sequential data, such as natural language, which cannot be applied to gene expression data [28,29]. In this study, a modified squeeze-and-excitation (SE) block [41] for gene expression data, namely the gene attention layer, is proposed. While the SE block is developed for a convolutional layer with three-dimensional image data, it is modified to be able to be used for one-dimensional gene expression data in this study. The proposed structure of the gene attention layer can be represented as follows:

(3) $$A≔Sigmoid(W_{GA-2}\cdot ReLU(W_{GA-1}\cdot X_{0})),$$

(4) $$X_{1}≔A^{T}⊙X_{0},$$

where $W_{GA-1}\in {\mathbb{R}}^{q\times p}$**,** $W_{GA-2}\in {\mathbb{R}}^{p\times q}$ are weight matrices of the gene attention layer in which p is the number of genes, ℝ is the real coordinate space, and q≪p; *ReLU* represents the rectified linear unit; $X_{0}$ is the gene expression; $X_{1}$ is the output of the gene attention layer, which is the input of the following deep learning structures. The dimension of the hidden layer in gene attention, i.e., q, is set to a significantly low value (<100), while the number of genes, i.e., p, is approximately 20,000 in RNA-seq data.

Because the attention vector A can have a value between 0 to 1 per gene, Equation (4) signifies that the gene expression data are not fully conveyed to the next layer. Specifically, a lower attention value for a gene regulates the gene; thereby, information in the gene is not further used in deep learning. The weight matrices in the gene attention layer $W_{GA-1}$ and $W_{GA-2}$ are trained during the training process in deep learning with the backpropagation algorithm. This process implies that the attention values are determined through training with data, not manually. Thus, the attention values are a result of the training and are not controllable by users.

Consequently, the attention value for a gene indicates how much the gene is significant in the deep learning model as well as the target of the model. Therefore, when the target of the model is the prognosis of a disease, the attention values can represent the prognostic genes for the disease. This factor of the gene attention layer is the main advantage of the attention mechanism in which significant features with respect to deep learning can be explained, while deep learning has been generally considered a black box.

### 2.2. Residual Deep Learning

Residual learning is a method to create a skip connection that links backward and forward of one or more layers [35]. Because gradients from the final layer of a deep learning model can also flow the skip connection, it is more appropriate to train initial layers that are far from the final layer. Thus, in recent years, the residual learning framework has become essential to train deep learning models since the method can alleviate the vanishing gradient problem, which has been a significant hindrance in deep learning. The structure of residual learning can be represented as follows:

(5) $$X_{k+m+1}≔X_{k}+\sigma_{k+m}(W_{k+m}\cdots \sigma_{k}(W_{k}\cdot X_{k})),$$

where m is the number of layers that the skip connection bypasses.

### 2.3. Layer Normalization

Layer normalization (*LN*) is a method to maintain the learning space of a layer [36]. During the training of a deep learning model, without a normalization method, the distribution of inputs of a layer fluctuates, where the means and variances of the distribution change in each iteration. Since the output of the layer becomes the input of the next layer, the fluctuation yields another instability in the next layer. Such instability is accumulated throughout a deep learning model and causes a problem to be handled.

Normalization methods are required between layers in a deep learning model to address this problem. These normalization methods maintain the means and variances of inputs during the training process and stabilize the deep learning training. Among the normalization methods, *LN* is employed in the proposed model in this study because gene expression data generally lack the number of samples (i.e., the number of patients) compared to other datatypes for deep learning training. Thus, the number of samples in a mini-batch should be less than that of conventional deep learning. When the number of samples in a mini-batch is small, it has been demonstrated that *LN* outperforms the batch normalization (BN) method [42]. The *LN* method used in this study can be computed as follows:

(6) $$LN\left(X_{k}\right)≔\frac{X_{k}-\mu }{\sigma },$$

(7) $$\mu =\frac{1}{q}\sum_{x\in X_{k}}x,\;\;\sigma =\sqrt{\frac{1}{q}\sum_{x\in X_{k}}{\left(x-\mu \right)}^{2}},$$

where q is the number of nodes in the layer. This method is a modified *LN* where adaptive parameters, i.e., gains and biases, are used in the conventional *LN*.

### 2.4. Ensemble Learning

Overfitting is one of the significant issues in the training of deep learning. Complex machine learning models, including deep learning, have more training parameters than the number of samples, which causes the overfitting problem. Machine learning models and deep learning models with overfitting produce accurate answers to training sets, while the performance in test sets is relatively inferior. The ensemble learning method has been used to alleviate this overfitting problem [43,44]. In ensemble learning, many models are trained and produce estimations. Then, the final estimation is made by a weighted sum or the average of the estimations by the models.

In this study, an ensemble of multiple deep learning models is employed. In deep learning, models can yield different results even if the same structure is used because the random initialization of weight parameters and mini-batch sampling cause such a difference in training results. Integrated with the overfitting problem, this inconsistency of training results is also one of the significant issues in deep learning. The ensemble learning can handle these problems since the noises from the overfitting and randomness are offset, and estimations are overlapped and added. The ensemble deep learning models used in this study yield the final result as follows:

(8) $$\hat{Y}≔\frac{1}{n}\sum_{i\in \left\{1,\ldots ,n\right\}}DL_{i}\left(X\right),$$

where n is the number of sub-models; $DL_{i}$ is ith sub-model. Each $DL_{i}$ has the identical structure, but the random initialization of weight parameters causes different training results and estimations.

### 2.5. The Gene Attention Ensemble Network

**Figure 1:**
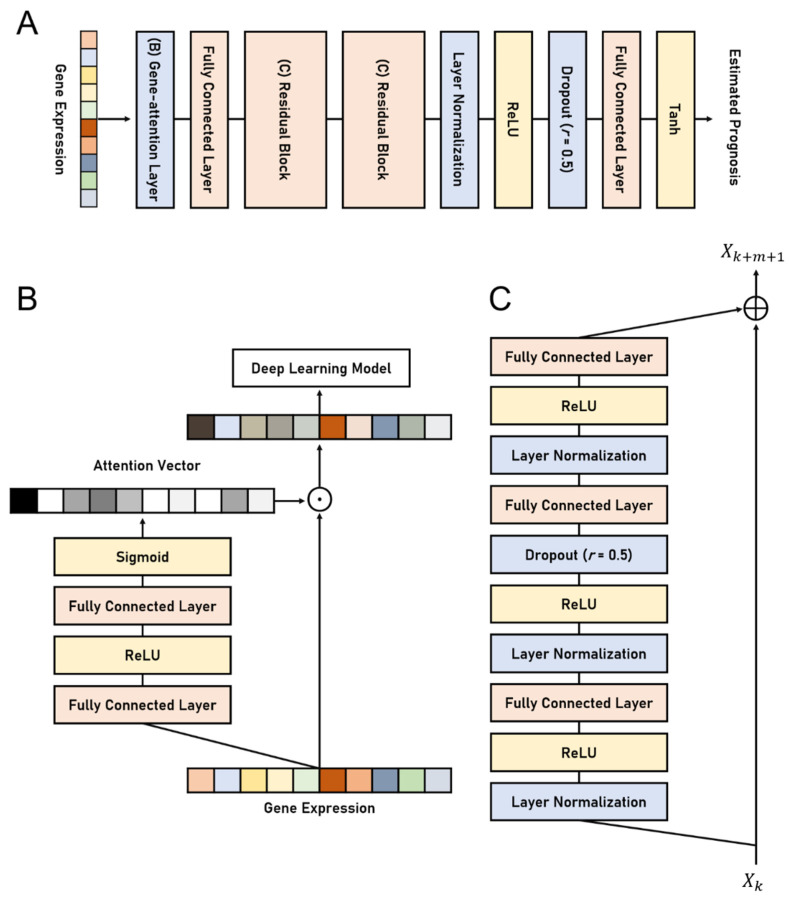
The architectures of the GAENET. (**A**) The architecture of a sub-model in the GAENET. (**B**) The proposed gene attention layer. (**C**) Residual block in the sub-model. The gene attention layer and residual blocks in (**A**) are illustrated in (**B**) and (**C**), respectively. Each purpose of the process is indicated by its color: Orange denotes a deep learning layer; yellow denotes an activation function; blue denotes regularization methods for layer outputs. The GAENET is composed of an ensemble of *n* number of the sub-models in (**A**), whereas the models share the gene attention layer. The *r* in the dropout layers indicates the dropout rate.

The proposed deep learning model, namely the Gene Attention Ensemble NETwork (GAENET), uses the proposed gene attention layer, residual learning, *LN*, and the ensemble structure. The proposed structures are illustrated in Figure 1. The residual learning is employed in the hidden layer of the GAENET, in which three fully connected layers are used in each residual block. *LN* is used between an activation function and a fully connected layer, which is a conventional method to use *LN*. As mentioned, the proposed gene attention layer becomes the first layer of the GAENET, thereby discovering prognostic genes. Dropout layers are introduced in the middle of the residual block as well as prior to the last fully connected layer to alleviate overfitting. Since the GAENET consists of an ensemble of sub-models, each of which is a deep learning model (Figure 1A), the estimation of the GAENET is the average of *n* number of sub-models, while they share the weights parameters in the gene attention layer.

During the training of the GAENET, an appropriate loss for the backpropagation algorithm to optimize the weights of the model is required. To estimate the prognosis, log values of survival days of samples become the target of the model. In order to train relative survival according to gene expression, the target value of a sample is determined considering relative survival days in a mini-batch of a single backpropagation iteration instead of targeting the absolute value of survival days. Then, the GAENET aims at minimizing the difference between estimation and the relative survival days. The loss to minimize the difference is computed as follows:

(9) $$L≔-\frac{1}{s_{m}}{\left(\log Y-\frac{1}{s_{m}}\sum_{i\in \left\{1,\ldots ,s_{m}\right\}}\log Y_{i}\right)}^{T}\left(\hat{Y}-\frac{1}{s_{m}}\sum_{i\in \left\{1,\ldots ,s_{m}\right\}}{\hat{Y}}_{i}\right),$$

where L is the loss to minimize; $s_{m}$ is the number of samples in a mini-batch; $Y_{i}$ and Y are the survival days of ith sample and the vector of survival days, respectively; $\hat{Y}$ represents the vector of prognosis estimations for a mini-batch. Since the output activation of the GAENET is the hyperbolic tangent, the model tries to estimate 1 if a sample has a higher survival day than the average of the mini-batch; otherwise, −1 is targeted. Therefore, the output value of a trained GAENET becomes low if the prognosis of the input sample is likely to be poor.

Thus, the GAENET is trained with survival days. By using the survival days as the target, these estimation values from −1 to 1 produced by the GAENET become correlated with survival days, since Equation (9) aims to maximize the correlation between survival days and the values. In this manner, it is possible to infer that a patient seems to have a poor prognosis if his or her gene expression has a lower estimation value approaching −1.

After the training of the GAENET, the attention vector in the gene attention layer signifies how much the genes are used to perform the estimation. Thus, the values of the attention vector indicate prognostic genes, where the prognostic genes have high attention values while the others have low values. In order to determine prognostic genes, attention vectors used for each sample are averaged as follows:

(10) $$P_{G}≔\frac{1}{s}\sum_{i\in \left\{1,\ldots ,s\right\}}GA\left(X^{\left(i\right)}\right),$$

where $P_{G}$ is the prognostic gene score; $X^{\left(i\right)}$ indicates gene expression of a sample; s is the number of samples; $GA\left(\cdot \right)$ is the sigmoid outputs in the gene attention layer.

### 2.6. Hyperparameters for Training and Experimental Settings

The number of sub-models in the GAENET, i.e., n in Equation (8), was set at 10. The number of nodes of the layers in the GAENET was 16; however, the number of nodes was reduced to 8 at the first and second layers in each residual block to alleviate overfitting. The number of nodes in the hidden layer of gene attention was set at 10. The Adam optimization algorithm [45] with a learning rate of 0.0001 was used to train the model. In the experiments, the model was trained with 100 epochs; 5 mini-batches were randomly composed for each epoch.

The model was evaluated using cross-validation with 20% of the samples as a test set. The test set was randomly selected from the samples in the dataset, and this cross-validation was performed 30 times with random test sets. The performance of the model was compared to conventional methods, ridge regression, survival support vector machine (SurvivalSVM) [46], random survival forest (RSF) [47], and Coxnet [48]. The performance was measured by C-Index and the log-rank test [49].

The Cancer Genome Atlas for Low-Grade Glioma dataset (TCGA-LGG) was used in the experiments. The uncensored 125 samples were employed for cross-validation; thus, 25 samples became a test set in each cross-validation. The RNA-seq data were normalized by RNA-Seq by Expectation-Maximization (RSEM) [50]; then, the gene expression data were transformed by log normalization. If the zero value of a gene exceeds 50%, the gene was excluded from the dataset.

## 3. Results

### 3.1. The Prognosis Estimation of the GAENET

**Figure 2:**
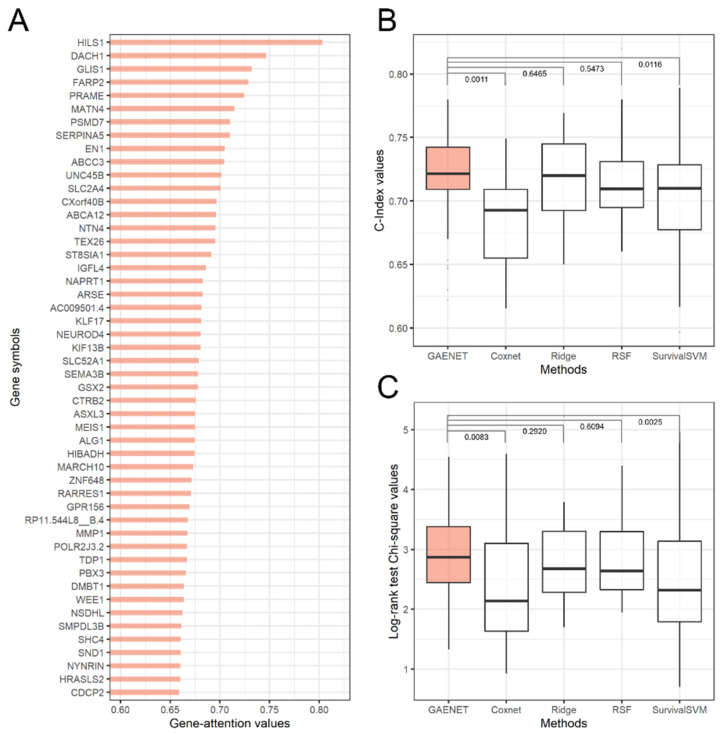
Prediction results of the GAENET. (**A**) Estimated prognostic genes for low-grade glioma. (**B**) Comparison of estimation performance with C-Index. (**C**) Comparison of estimation performance with the log-rank test. The estimated prognostic genes were discovered with test sets. The values above the boxplots are the Wilcoxon signed rank test *p*-values. The results of log-rank test are exhibited with square root values of Chi-square values. The estimation performances were measured with 30 times of cross-validations. The proposed gene attention algorithm ranked *HILS1*, a pseudogene, as the most significant prognostic gene. The log-rank tests indicated that the proposed GAENET had a median χ value of 2.870, a difference of 7.2 percent from the second-best model, ridge regression.

The performance comparison and the top-50 prognostic genes of LGG estimated by the GAENET are shown in Figure 2. As a result, *HILS1* was estimated to be the most significant prognostic gene with an attention value of 0.8033. While the linear relationship between genes and prognosis has conventionally been studied, deep learning models can estimate the prognosis in a nonlinear manner. This indicates that the prognostic genes estimated by the GAENET are significant considering combinations with the expression of other genes. Since genes regulate and promote with each other by composing gene networks, such combinational effects can be noticeable in estimating prognosis.

*HILS1* has been reported as a pseudogene [51], which is considered that it is not likely encoded to a protein. However, recent studies have demonstrated that some pseudogenes regulate the expression of other genes, verifying the relationships with the prognosis of cancers [52]. In addition, previous studies have reported that *HILS1* is a prognostic gene of LGG, considering the linear relationships with the prognosis [53]. In addition, the second and third significant prognostic genes, *DACH1* and *GLIS1*, have been widely reported that they are associated with the regulation of tumors and cancers [54,55]. Conversely, it has not yet been reported whether *FARP2*, the fourth prognostic gene, is associated with LGG, which should be further studied in terms of nonlinear and combinational perspectives with other genes since the prognostic genes estimated by the GAENET are selected in such a view.

In the performance evaluation, the GAENET outperformed the other methods, considering C-Index and Chi-square values. For example, the GAENET exhibited a median χ value of 2.870, demonstrating a 7.2% difference compared to the second-best model, ridge regression. As shown in the Figure 2, it should be noted that the variance of performance of the GAENET is relatively low, which is one of the advantages of ensemble structures.

### 3.2. Combinational Analysis of Estimated Prognostic Genes

As mentioned in the previous section, the estimated prognostic genes were discovered from a perspective of deep learning, which was nonlinear and combinational. Such combinational effects are significant, but prognosis has conventionally been analyzed in a single gene with a linear relationship considering the nature of gene expression. In this study, a combinational analysis of gene expression with the estimated prognostic genes was performed. The gene expression of two groups, poor prognosis and good prognosis, were analyzed. The good prognosis group and the poor prognosis group are in the first quartile and the fourth quartile of survival days, respectively. The maximum survival of the poor prognosis group was 438 days, while the minimum survival of the good prognosis group was 1578 days.

**Figure 3:**
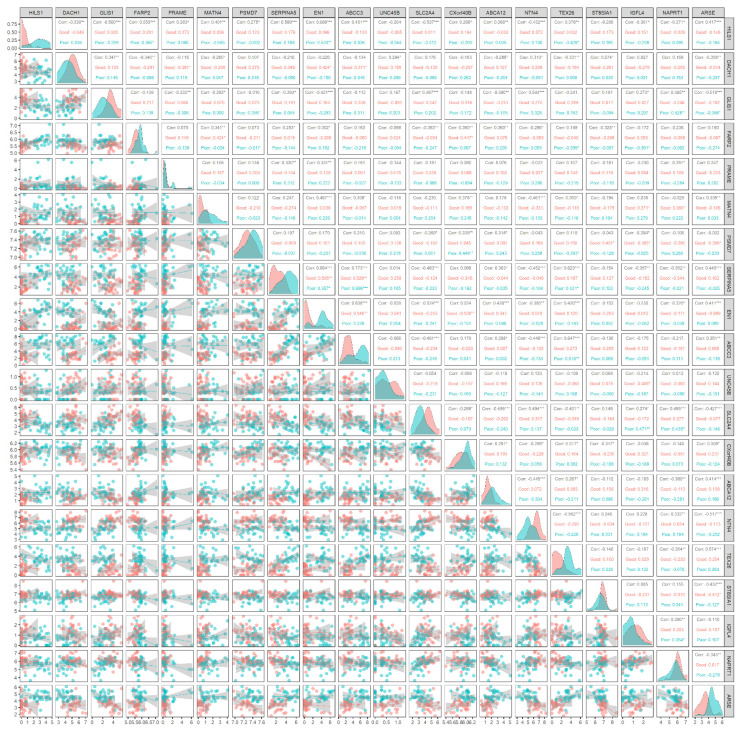
Joint distributions of gene expression of the estimated prognostic genes. The good prognosis group and the poor prognosis group are in the first quartile and the fourth quartile of survival days, respectively. Diagonal elements represent the marginal distribution of each gene. Lower-triangle elements exhibit the sample distribution for the groups. Upper-triangle elements display the correlation in each group. In the correlation, the symbols *, **, and *** indicate *p* < 0.05, < 0.01, and < 0.001, respectively. It is established that the expression of *HILS1* is correlated with the expression of other prognostic genes; 17 genes are correlated with *HILS1* (*p* < 0.05), while 8 genes are highly correlated (*p* < 0.001), implying that *HILS1* is a biomarker likely to regulate the expression of other prognostic genes.

The gene expression of the prognostic genes according to the group was distinct from each other. As shown in Figure 3, the gene expression of two groups with respect to *HILS1* was noticeable; in the poor prognosis group, *HILS1* was up-regulated while it was down-regulated in the other group. In addition, it is demonstrated that the expression of *HILS1* is correlated with that of the other prognostic genes; among the 19 genes, it is confirmed that 17 genes are correlated (*p*-value < 0.05) with *HILS1* while eight genes are highly correlated (*p*-value < 0.001), which implies that *HILS1* is a biomarker likely to regulate the expression of other prognostic genes. This factor accords with the characteristic of *HILS1*, in which a pseudogene is not encoded into a protein while they may regulate other genes [56].

The most significant correlation was observed between *ABCC3* and *SERPINA5*; they were commonly up-regulated in the poor prognosis group. The joint distribution of gene expression exhibited that *TEX26* and *ABCC3* are correlated (*p*-value < 0.01) only in the poor prognosis group, whereas no correlation is observed in the other group. This implies that these two genes may jointly affect the poor prognosis of LGG. Such correlations only in the poor prognosis group (*p*-value < 0.01) were also observed between *HILS1* and *EN1*, *GLIS1* and *NAPRT1*, *PSMD7* and *CXorf40B*, and *SLC2A4* and *IGFL4*.

The most important finding of this analysis is that *HILS1* expression is substantially linked with the expression of other prognostic genes. This suggests that *HILS1* may regulate other prognostic genes, resulting in prognostic disparities. As a result, *HILS1* is a likely candidate for down-regulation, given its high expression in patients with a poor prognosis. Additionally, some genes were shown to be correlated exclusively in the group with a poor prognosis but not in the other group. This implies that a few genes identified in this investigation may contribute to the poor prognosis of LGG in combination.

### 3.3. Ablation Study of the GAENET

**Figure 4:**
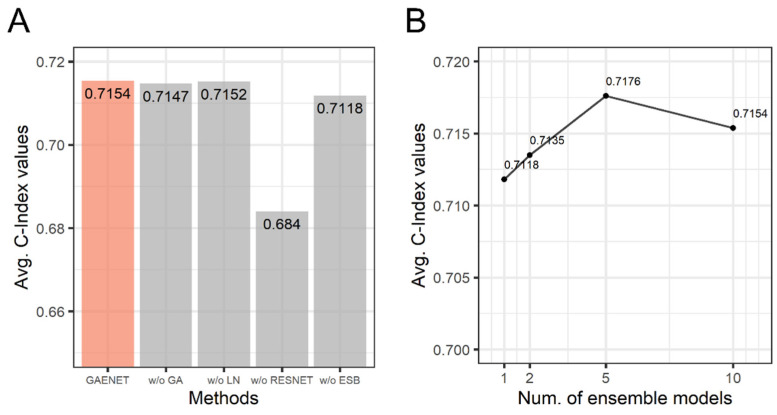
The results of ablation study of the GAENET. (**A**) C-Index values without each method. (**B**) C-Index values with respect to number of sub-models in the ensemble of the GAENET. GA: gene attention; LN: layer normalization; RESNET: residual learning; ESB: ensemble learning.

An ablation study was performed to investigate the effects of the methods used in the GAENET. In the ablation study, the performance without each method consisting of the GAENET was measured with C-Index. The models, each of which does not use one of gene attention, *LN*, residual learning, and ensemble learning, were compared. Additionally, the performance considering sub-models in the ensemble in the GAENET was analyzed. The performance is summarized in Figure 4.

As a result, while the GAENET showed the best performance, the models without gene attention and *LN* were marginally inferior to the GAENET. Conversely, the residual learning exhibited a significant difference in performance where the difference of C-Index was $3.14\times 10^{-2}$, which indicated the effectiveness of the residual learning in terms of the deep architecture of neural networks. Similarly, the performance was inferior to the GAENET when a single model was used instead of the ensemble in the GAENET.

As shown in Figure 4B, all ensemble models demonstrated superior performances compared to the single model. Additionally, the best performance (C-Index: 0.7176) was observed when the number of models was five. Although it was expected that the performance increased as the number of models increased, the GAENET with ten sub-models was marginally inferior to five sub-models due to various random effects in deep learning, such as the random initialization of weight parameters and random mini-batch sampling.

This result indicates that each of the deep learning techniques employed in the GAENET contributes to the performance of the model. Among the techniques, residual learning and ensemble learning are the most effective methods for dramatically improving performance. As a result, in this study, it is demonstrated that modern deep learning techniques can be adapted for gene expression and are more successful than ordinary deep learning models.

## 4. Discussion

A deep learning model for prognosis estimation with gene expression, the GAENET, and gene attention was proposed in this study. While other studies have employed ordinary deep learning models, i.e., MLP and AE, without recent developments [21,22], the GAENET demonstrated that the recent developments, such as attention mechanism and residual learning, can be modified for gene expression and exhibited superior performances compared to the models without the developments.

A few biomedical findings were exhibited by the proposed gene attention since gene attention can find prognostic genes through deep learning training. The proposed gene attention is informative in a nonlinear view because prognostic genes are considered with combinational effects and polynomial relationships. While conventional studies commonly focused on linear relationships with linear models, such as the Cox proportional-hazards model and Kaplan-Meier estimation, this study proposed a new method to investigate prognostic genes in a nonlinear view. As a result, *HILS1* was found as the most significant prognostic gene. This result accorded with other studies that reported *HILS1* was likely correlated with the prognosis of LGG [53].

Other studies have reported several prognostic genes of LGG. For instance, a study investigated the relationships between pseudogenes and the prognosis of LGG and reported *PKMP3*, *AC027612.4*, *HILS1*, *RP5-1132H15.3*, and *HSPB1P1* as candidates of prognostic pseudogenes of LGG [53]. A meta-analysis has found that high expression of *HIP1R* and low expression of *TBXAS1* were correlated with increased overall survival of diffuse LGG after investigating eight independent microarray datasets [57]. A recent study using the Cox model reported that *CTSZ*, *EFEMP2*, *ITGA5*, *KDELR2*, *MDK*, *MICALL2*, *MAP2K3*, *PLAUR*, *SERPINE1*, and *SOCS3* were related to the prognosis of glioblastoma multiforme and LGG, while the same dataset (TCGA) was used in this study [58].

Since these studies were based on the linear relationships between gene expression and prognosis as well as univariate analyses, the prognostic genes in these studies can be different from the result of gene attention. However, the most significant prognostic gene estimated by gene attention, i.e., *HILS1*, was also reported in the other study [53], which indicated that *HILS1* was a distinct prognostic gene not only in a nonlinear view but also in a linear view. 

Generally, *IDH1* mutation and 1p/19q codeletion has been known as prognostic biomarkers in glioma [59]. Since RNA-seq gene expression data are used in this study, the GAENET has a limitation in that the model is not able to detect such biomarkers. However, it has been reported that nearly all 1p/19q codeleted glioma has *IDH1* mutation [60]; therefore, such biomarkers can be represented by the gene expression of *IDH1* in a mediate manner, although the mutation cannot be directly detected. As a result, the gene attention found that *IDH1* was the top 2.2% prognostic gene, which was 443rd out of 19,990 genes. Given that the expression of *IDH1* was investigated instead of its mutation, it can be said that gene attention partially considered *IDH1* as a potential prognostic gene.

## 5. Conclusions

In this study, an ensemble deep learning model, namely the GAENET, was proposed to estimate the prognosis of LGG with gene expression. The GAENET employed the proposed attention mechanism for gene expression data, called gene attention. Owing to the advantage of the gene attention mechanism, it is possible to discover prognostic genes in a nonlinear and combinational perspective, which is the nature of deep learning training. While deep learning training has been considered a black box, it was demonstrated that prognostic genes in such a view can be determined by the gene attention layer. Additionally, a few conventional training methods for deep learning, including residual learning, were introduced.

As a result, it was demonstrated that the GAENET outperformed conventional methods for estimating prognosis, exhibiting a 7.2% performance difference compared to the second-best model. Additionally, the prognostic genes of LGG, including *HILS1*, *DACH1*, and *GLIS1*, were discovered by the proposed gene attention. In the ablation study of the GAENET, it was confirmed that the methods used in the GAENET contributed to the performance of the GAENET, in which the residual learning showed the most significant difference.

## Data Availability

The dataset used in this paper is a public dataset, obtained at Genomic Data Commons Data Portal: https://portal.gdc.cancer.gov/ (accessed on 27 February 2022).

## Appendix A

A deep learning model consists of weight matrices and nonlinear activation functions. Given an input, a deep learning model produces an output with a mathematical computation with the weight matrices and activation functions. The simplest deep learning model has a weight matrix and a nonlinear function, constituting a layer. In this model, the mathematical computation is simply multiplying the input and the weight matrix and applying the nonlinear function, which can be represented as follows:

(A1) $$\hat{Y}≔\sigma \left(\left[\begin{array}{ccc} w_{11} & \cdots & w_{1j}\\ \vdots & \ddots & \vdots \\ w_{i1} & \cdots & w_{ij} \end{array}\right]\cdot \left[\begin{array}{c} x_{1}\\ \vdots \\ x_{j} \end{array}\right]\right),$$

where the matrix with elements of w is the weight matrix; the vector with elements with x is the input; σ is the activation function.

This computation in Equation (A1) produces an output with i number of elements. Such a computation can be repeated by treating the output as a new input for the next layer with a different weight matrix and activation function. The number of repetitions indicates the depth of the model. In this manner, Equations (1) and (3) are deep learning models with multiple layers.

Modifications of Equation (A1) can also be a layer of deep learning models. Numerous modifications for each purpose have been proposed. Equations (2) and (4) regularize the output of a layer by multiplying specific values. When smaller values are multiplied, the resulting outputs become less significant, whereas outputs multiplied by larger values become critical. These multiplied values are also defined by a deep learning model of Equation (3), which is the proposed gene attention layer.

Additionally, Equations (5)–(7) correspond to the modifications necessary for optimal deep learning model training. The residual learning in Equation (5) is for backpropagation with smooth gradient flows. The layer normalization in Equations (6) and (7) also correspond to modifications of layer outputs, as in Equations (2) and (4). It maintains output distributions for more effective training.
